# Alcalase-Based Chickpea (*Cicer arietinum* L.) Protein Hydrolysates Efficiently Reduce Systolic Blood Pressure in Spontaneously Hypertensive Rats

**DOI:** 10.3390/foods13081216

**Published:** 2024-04-16

**Authors:** Oscar Gerardo Figueroa-Salcido, Jesús Gilberto Arámburo-Gálvez, José Antonio Mora-Melgem, Diana Laura Camacho-Cervantes, Martina Hilda Gracia-Valenzuela, Edith Oliva Cuevas-Rodríguez, Noé Ontiveros

**Affiliations:** 1 Integral Postgraduate Program in Biotechnology, Faculty of Chemical and Biological Sciences, Autonomous University of Sinaloa, Ciudad Universitaria, Culiacan 80010, Sinaloa, Mexico; oscar.figueroa@uas.edu.mx; 2 Nutrition Sciences Postgraduate Program, Faculty of Nutrition Sciences, Autonomous University of Sinaloa, Culiacan 80019, Sinaloa, Mexico; gilberto.aramburo@uas.edu.mx (J.G.A.-G.); josemora.uacng@uas.edu.mx (J.A.M.-M.); dianacamacho.uacng@uas.edu.mx (D.L.C.-C.); 3 National Technological Institute of Mexico, Yaqui Valley Technological Institute, Bácum 85276, Sonora, Mexico; martina.gv@vyaqui.tecnm.mx; 4 Clinical and Research Laboratory (LACIUS, C.N.), Department of Chemical, Biological, and Agricultural Sciences (DC-QB), Faculty of Biological and Health Sciences, University of Sonora, Navojoa 85880, Sonora, Mexico

**Keywords:** ACE-I, chickpea, hypertension, hydrolysate, bioactive peptides, antihypertensive peptides

## Abstract

Studies on antihypertensive chickpea protein hydrolysates have rarely performed in vivo evaluations, limiting the entry of such hydrolysates into functional food development and clinical trials. Thus, our aim was to optimize the hydrolysis conditions to produce an alcalase-based chickpea hydrolysate with a hypotensive effect in vivo at convenient oral doses. The hydrolysis reaction time, temperature, and alcalase/substrate concentration were optimized using a response surface analysis (RSA). ACE-I inhibition was the response variable. The optimized hydrolysis conditions were time = 0.5 h, temperature = 40 °C, and E/S concentration = 0.254 (U/g). The IC_50_ of the optimized hydrolysate (OCPH) was 0.358 mg/mL. Five hydrolysates from the RSA worksheet (one of them obtained after 5 min of hydrolysis (CPH15)) had an ACE-I inhibitory potential similar to that of OCPH (*p* > 0.05). At 50 mg/kg doses, OCPH and CPH15 promoted a clinically relevant hypotensive effect in spontaneously hypertensive rats, up to −47.35 mmHg and −28.95 mmHg, respectively (*p* < 0.05 vs. negative control). Furthermore, the hypotensive effect was sustained for at least 7 h post-supplementation. Overall, OCPH and CPH15 are promising ingredients for functional food development and as test materials for clinical trials.

## 1. Introduction

Hypertension is a chronic condition and the major risk factor for developing cardiovascular diseases. Around 31.1% of the adult population has hypertension and consequently an increased risk of premature death [1]. Untreated hypertensive individuals have persistent high levels of systolic (≥140 mmHg) and diastolic (≥90 mmHg) blood pressure, and most of them (around 90%) develop primary or essential hypertension, the pathogenesis of which involves environmental and genetic factors [2,3]. Certain molecules associated with the renin–angiotensin–aldosterone system (RAAS) are therapeutic targets for the control of hypertension [4]. In fact, antihypertensive drugs like captopril, enalapril, and lisinopril inhibit the angiotensin-I-converting enzyme (ACE-I; peptidyldipeptide hydrolase, EC 3.4.15.1), which promotes the production of the potent vasoconstrictor angiotensin II (DRVYIHPF) and hydrolyzes the vasodilator bradykinin [5]. However, synthetic ACE-I inhibitors can trigger adverse reactions such as dry cough, dizziness, hyperkalemia, and hypotension, among others [6]. Therefore, there is interest in developing side-effect-free products for the treatment of hypertension.

Bioactive peptides have a wide spectrum of health-promoting properties, such as antioxidant, antidiabetic, antimicrobial, and antihypertensive [7]. Particularly, legumes are recognized as sources of bioactive peptides [8], and recent findings highlight that antihypertensive peptides can be derived from chickpea (*Cicer arietinum* L.) [9,10,11]. In this context, protein hydrolysates can be a reliable source of ingredients for the development of functional foods or be the base for well-tolerated peptide-based adjunct therapies. Recently, we demonstrated that a sequentially digested chickpea protein hydrolysate had antihypertensive properties [9]. However, the conditions for obtaining an antihypertensive hydrolysate with clinically relevant potential at convenient oral doses remain to be optimized. Chickpea is the third most cultivated legume around the world and contains a high protein content (20–22%), making it suitable for the production of natural functional ingredients [12]. Therefore, our aim was to obtain an optimized chickpea protein hydrolysate that could reduce systolic blood pressure in spontaneously hypertensive rats after intragastric supplementation.

## 2. Materials and Methods

### 2.1. Obtention of the Chickpea Protein Isolate

**Figure 1:**
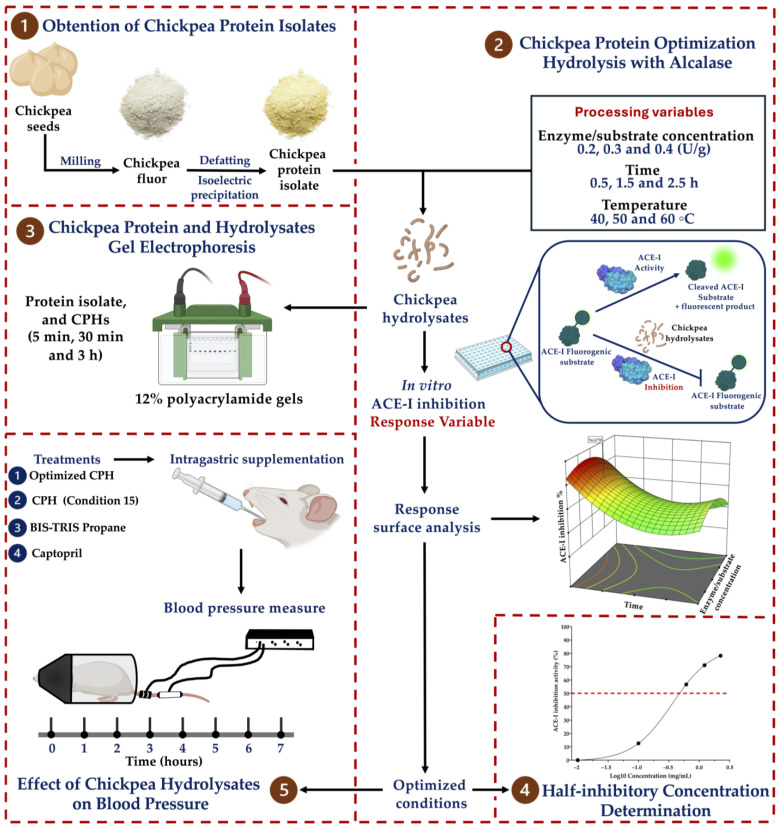
General workflow employed to assess the ACE-I inhibitory potential and antihypertensive effect of chickpea hydrolysates. Numbers indicate the order of the analyses performed. Acronyms. CPH, chickpea protein hydrolysate. Condition 15: temperature = 50 °C, time = 0.0896 h (5 min), and E/S concentration = 0.3 U/g.

Figure 1 outlines the overall methodology employed to assess the antihypertensive potential of the chickpea protein hydrolysates. Chickpea seeds (Blanco Sinaloa 92, Granos La Macarena^TM^, Navojoa, Mexico) were milled using a Model 4 Wiley^®^ Laboratory Mill (Thomas Scientific, Swedesboro, NJ, USA) to obtain the flour. The chickpea protein was extracted according to Chávez-Ontiveros et al. 2022 with minor modifications [9]. Briefly, the chickpea flour was defatted using acetone (1:4 *w/v*). The samples were stirred at 500 rpm for 4 h and dried overnight at room temperature (25 °C). The defatted flour was resuspended in distilled water (1:10 *w/v*), the pH of the suspension was adjusted to 8.5 using NaOH 1 M, and it was stirred at 500 rpm for 2 h. The suspension was centrifuged at 10,000× *g* for 10 min, the supernatant was collected, and the pellets were washed once using the same conditions. Afterward, the supernatants collected were combined, and the pH of the solution was adjusted to 4.5 (HCI 1 M) to initiate protein precipitation. The precipitation was carried out for 2 h with constant stirring at 500 rpm. The solutions were centrifuged at 10,000× *g* for 10 min, and the pellets were collected, lyophilized, and stored at −20 °C until their use. Their protein content was determined using the micro-Kjeldahl assay (AOAC method 960.52). The extraction yield and protein recovery yield were calculated using the following equations [13]:

$$Extraction\;yield\left(\%\right)=\frac{Weight\;of\;lyophilized\;powder\left(g\right)}{Weight\;of\;deffatted\;chickpea\;flour\left(g\right)}\times 100$$

$$Protein\;recovery\;yield\left(\%\right)=\frac{Protein\;content\;in\;lyophilized\;powder\left(g\right)}{Protein\;content\;indeffatted\;chickpea\;powder\left(g\right)}\times 100$$

### 2.2. Chickpea Protein Optimization Hydrolysis with Alcalase

A response surface analysis (RSA) with a central composite design was performed. A worksheet with randomly selected hydrolysis reactions was generated. The hydrolysis was carried out using alcalase (≥2.4 U/g, Sigma-Aldrich, St. Louis, MO, USA). The experimental design is shown in Table 1. The following processing variables were optimized: (1) enzyme/substrate concentration (U/g), (2) time (h), and (3) temperature (°C). Three levels were considered for each variable. The central conditions were taken from a previous study with minor modifications to expand the experimental region [14]. ACE-I inhibition was the response variable.

Fifteen mg of the chickpea protein isolate was solubilized in 1 mL of BIS-TRIS Propane buffer (20 mM; pH 11) (Sigma-Aldrich, St. Louis, MO, USA). Afterwards, the samples were sonicated for 10 min (Ultrasonic Homogenizer Model 150 V/T; power = 40; pulser = 30%) and shaken at 1000 rpm (50 °C) for another 10 min. Before the addition of alcalase (≥2.4 U/g, Sigma-Aldrich, St. Louis, MO, USA), the temperature of the solution was adjusted according to the experimental design (Supplementary Materials Table S1). The hydrolysis reactions were carried out using a Thermomixer (Eppendorf™, Hamburg, Germany), and the reactions were stopped by heating the samples at 85 °C for 15 min. Finally, the samples were stored at −20 °C until their use.

### 2.3. Determination of the ACE-I Inhibition of the Chickpea Hydrolysates

The ACE-I inhibition percentage was determined using the ACE-I activity kit (Sigma-Aldrich CS0002, Saint Louis, MO, USA). This assay is based on the capacity of ACE-I to hydrolyze a fluorogenic substrate, resulting in a fluorescent product. The measured fluorescence is proportional to the ACE-I activity. Briefly, the ACE-I and fluorogenic substrate reagents were diluted in assay buffer following the manufacturer’s instructions. A total of 10 μL of chickpea protein hydrolysate (CPH) (10 mg/mL) was added to all the wells except the positive control (10 μL of BIS-TRIS Propane buffer (20 mM)). Afterwards, 5 μL of ACE-I and 40 μL of assay buffer were added to all the wells. Finally, 50 μL of the fluorogenic substrate was added, and the reactions were carried out at 37 °C. The ACE-I activity was measured using a fluorescent plate reader (Varioskan™ LUX, Thermo Scientific, Waltham, MA, USA) with excitation and emission wavelengths of 320 and 405 nm, respectively. The area under the curve was calculated considering the reaction time kinetics, where the positive control reached 100% ACE-I activity. The ACE-I inhibition percentage was determined as follows:

$$ACE-I\;Inhibition\left(\%\right)=\frac{A-B}{A}\times 100$$

where A = the area under the curve of the positive control (reaction without inhibitor) and B = the area under the curve of the samples.

### 2.4. Experimental Validation of the Response Surface Analysis

Chickpea protein was hydrolyzed using the predicted (optimized) parameters, and the ACE-I inhibition percentage was estimated to validate the model. Three independent experiments were evaluated in duplicate for this purpose. To compare the experimental ACE-I inhibition data with the predicted data, the residual standard error (RSE) was calculated using the following equation:

$$RSE\left(\%\right)=\frac{Experimental\;value-Predicted\;value}{Predicted\;value}\times 100$$

### 2.5. Half-Inhibitory Concentration

The half-inhibitory concentration (IC_50_) of the optimized chickpea protein hydrolysate (OCPH) was determined using 5-point non-linear regression. The protein concentration was determined using the Bradford assay (Bio-Rad Protein Assay) and a bovine serum albumin standard curve. The OCPH concentration required to produce 50% ACE-I inhibition was defined as IC_50_ (mg/mL).

### 2.6. Chickpea Protein and Hydrolysate Gel Electrophoresis

The electrophoretic profile of the chickpea proteins and hydrolysates was determined using SDS-PAGE. Commercially available 12% polyacrylamide gel (Mini-PROTEAN^®^ TGX Stain-Free, Bio-Rad, Hercules, CA, USA) and protein molecular weight markers from 250 to 10 kDa (Bio-Rad, Cat. 161-0363) were used. The electrophoretic profiles of the chickpea protein isolate and chickpea protein hydrolysates (CPHs) generated after 5 min, 30 min, and 3 h of hydrolysis were determined. The lanes were loaded with 7.5 mg/mL and 15 mg/mL of protein. The protein bands were visualized using the ChemiDoc Imaging System (Bio-Rad) and analyzed using Image Lab^TM^ version 5.2.1 software (Bio-Rad, Hercules, CA, USA).

### 2.7. Animals

Male spontaneously hypertensive rats (SHRs) (12–16 weeks old, 250–300 g body weight) were used for the blood pressure assays. The SHRs were obtained from the National Autonomous University of Mexico (UNAM, Cell Physiology Institute) and placed in plastic cages with stainless steel lids. The room temperature was maintained at 24 °C with 12 h light/dark cycles. Food (LabDiet^®^ 5001, Richmond, IN, USA) and water were available ad libitum.

### 2.8. Effect of the Chickpea Hydrolysates on Blood Pressure

Blood pressure was evaluated in seven SHRs. The hydrolysates were administered at 50 mg/kg of body weight. BIS-TRIS Propane buffer (20 mM) and captopril (25 mg/kg of body weight) were used as the negative and positive controls, respectively. All the treatments were administered intragastrically using sterile plastic feeding tubes (18 GA × 75 mm, Instech Laboratories, Inc., Plymouth Meeting, Montgomery, PA, USA). Wash-out periods of at least 48 h were implemented between treatments. Their systolic blood pressure (SBP) was measured at 0 h (before treatment) and 1, 2, 3, 4, 5, 6, and 7 h (after treatment) using a CODA tail cuff blood pressure monitor.

### 2.9. Statistical Analysis and Ethical Aspects

The Shapiro–Wilk and Barlett’s tests were used to assess the data distribution and homoscedasticity, respectively. The data were expressed as the mean and standard deviation. Differences in protein content were assessed using an unpaired *t*-test. Differences in SBP among the treatments and ACE-I inhibition among the CPHs were assessed using factorial ANOVA and Brown–Forsythe ANOVA, respectively. Multiple comparisons were determined using the two-stage linear step-up procedure of Benjamini, Krieger, and Yekutieli. The statistical analyses were performed using GraphPad Prism 9.0 (GraphPad Software, San Diego, CA, USA). The RSA was performed using Design-Expert software 11.0 (Stat-Ease, Inc., Minneapolis, MN, USA). A *p*-value < 0.05 was considered statistically significant. The study was conducted per the Declaration of Helsinki, and the experimental protocol was approved by the Ethics Committee of the Autonomous University of Sinaloa (CE-UACNYG-2015-SEP-001).

## 3. Results

### 3.1. Protein Content and Optimization of the Chickpea Hydrolysate

The protein content of the chickpea flour and the protein isolate obtained was 25.74 ± 1.99% and 94.76 ± 3.25% (*p* < 0.0001 vs. flour), respectively. The extraction and protein recovery yields were 17.58 ± 0.52% and 64.73 ± 1.94%, respectively. This protein isolate was used for the RSA analysis, which generated 33 experiments.

The effects of different chickpea protein hydrolysis conditions on the ACE-I inhibition are shown in Table S1. The ANOVA results determining the significance of the linear, quadratic, and interaction terms of the model are shown in Table S2. The model significantly influences the response variable (*p* < 0.0001), and the time contributes linearly (A) and quadratically (A^2^) to ACE-I inhibition (*p* < 0.05). The enzyme/substrate concentration (B) has a non-significant linear effect on ACE-I inhibition (*p* > 0.05), but it has a significant quadratic effect (B^2^) and significant interactions with the response variable in combination with temperature (BC) (*p* < 0.05). Regarding temperature, it has a significant linear effect (C) on ACE-I inhibition (*p* < 0.05).

**Figure 2:**
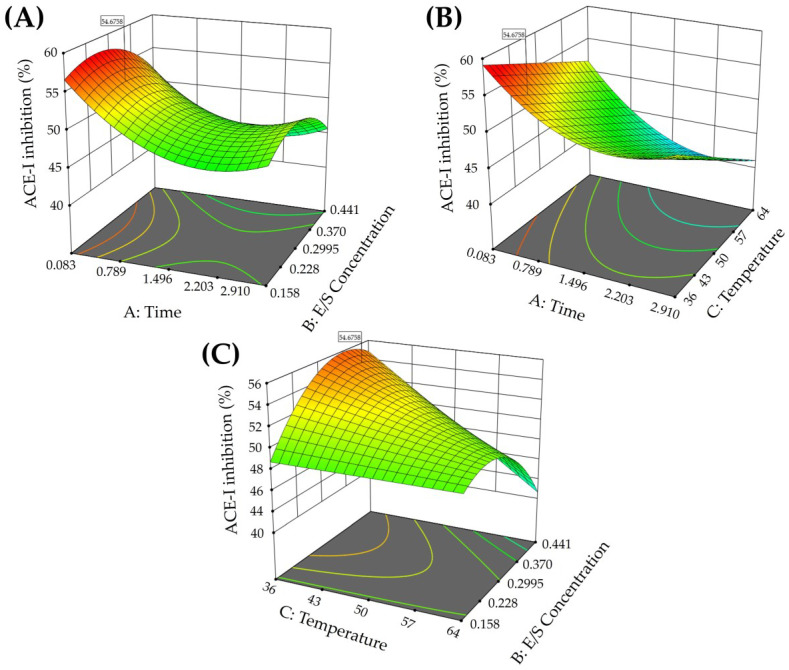
Response surface plots of the effects on ACE-I inhibition. (**A**) Effect of the interaction of time and enzyme/substrate concentration on ACE-I inhibition; (**B**) effect of the interaction of time and temperature on ACE-I inhibition; (**C**) effect of the interaction of temperature and enzyme/substrate concentration on ACE-I inhibition.

The coefficient of determination (R^2^) and the adjusted coefficient of determination were 0.79 and 0.72, respectively. Thus, the model explains 79% of the variability observed in ACE-I inhibition. The lack of fit was non-significant (*p* = 0.1674), which suggests that the model can be used to fit the effect of the process variables on ACE-I inhibition. The adequate precision (12.58) and the coefficient of variation obtained (5.21%) indicate that the model shows good consistency and accuracy and it can predict the values in the experimental region. The relationship between the process variables and ACE-I inhibition is shown in Figure 2. Higher hydrolysis reaction times were related to decreased ACE-I inhibition (Figure 2A,B). Contrary to this, lower hydrolysis reaction temperatures showed increased ACE-I inhibition (Figure 2B,C).

The RSA equation for calculating the maximum ACE-I inhibition theoretical value was as follows:

(1) $$\begin{array}{c} ACE-Iinhibition\left(\%\right)=+74.52944-8.09900\left(A\right)+22.41651\left(B\right)-\\ 0.657319\left(C\right)+1.94518\left(AB\right)-0.047290\left(AC\right)+1.55219\left(BC\right)+\\ 2.30543\left(A^{2}\right)-168.22346\left(B^{2}\right) \end{array}$$

where A = time (h), B = enzyme/substrate concentration (U/g), and C = temperature (°C). The maximum percentage of ACE-I inhibition predicted was 54.67% (95% CI = 52.21–57.13), considering the following conditions: time = 0.5 (h), enzyme/substrate concentration = 0.254 (U/g), temperature = 40 °C. Experimental validation of the model using the predicted conditions showed an ACE-I inhibition of 56.26 ± 0.85%. The RSE of the experimental validation was 2.90%.

### 3.2. ACE-I Inhibition of the Chickpea Hydrolysates and IC_50_

**Figure 3:**
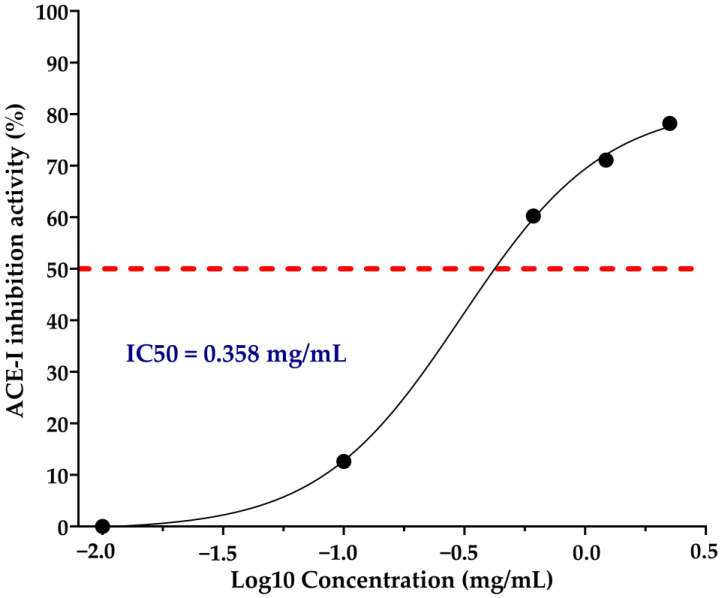
Half-inhibitory concentration of optimized chickpea protein hydrolysate.

The RSA generated 15 hydrolysis conditions. The ACE-I inhibition percentages of these CPHs ranged from 39.03 ± 3.31% to 55.69 ± 3.14 (Table 2). OCPH had the highest ACE-I inhibition percentage (56.26 ± 0.85%), which was significantly higher than 10 out of 15 hydrolysis conditions evaluated (*p* < 0.05) (Table 2). The CPH14 and CPH15 hydrolysates inhibited ACE-I at a similar percentage as OCPH did (*p* > 0.05), showing ACE-I inhibitions of 54.79 ± 2.10 and 55.59 ± 3.14%, respectively (Table 2). The IC_50_ of OCPH was 0.358 mg/mL (Figure 3).

### 3.3. Electrophoretic Characterization of the Chickpea Proteins and Hydrolysates

**Figure 4:**
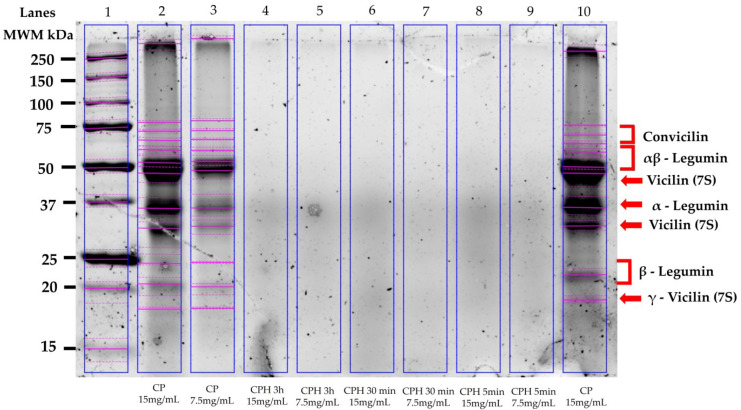
Gel electrophoresis of chickpea proteins (CPs) and chickpea protein hydrolysates (CPHs). Lane 1: molecular weight markers (MWM); lane 2: CP (15 mg/mL): lane 3: CP (7.5 mg/mL); lane 4: CPH generated after 3 h of hydrolysis (15 mg/mL; temperature = 50 °C and enzyme/substrate concentration = 0.3 U/g); lane 5: CPH generated after 3 h of hydrolysis (7.5 mg/mL; temperature = 50 °C and enzyme/substrate concentration = 0.3 U/g); lane 6: CPH generated after 30 min of hydrolysis (15 mg/mL; OCPH); lane 7: CPH generated after 30 min of hydrolysis (7.5 mg/mL; OCPH); lane 8: CPH generated after 5 min of hydrolysis (15 mg/mL; CPH15); lane 9: CPH generated after 5 min of hydrolysis (7.5 mg/mL; CPH15); line 10: CP (15 mg/mL). The software detected the bands by default and highlighted them with pink lines.

Figure 4 shows the electrophoretic patterns of the chickpea proteins and CPHs generated after 5 min, 30 min, and 3 h of hydrolysis with alcalase. Eleven bands were observed in the chickpea protein samples. The molecular weights were between 18 and 250 kDa. Three chickpea protein fractions were identified: convicilin (~70 kDa), legumin (αβ subunit = ~60 kDa; α subunit = 40 kDa; β subunit = 20 kDa), and vicilin (~45 kDa; ~35 kDa; ~18 kDa) (Figure 4, lanes 2, 3, and 10). Contrary to this, no bands were observed in the lanes loaded with the CPHs (5 min, 30 min and 3 h) (Figure 4, lanes 4–9).

### 3.4. Chickpea Hydrolysates Efficiently Reduce Systolic Blood Pressure

**Figure 5:**
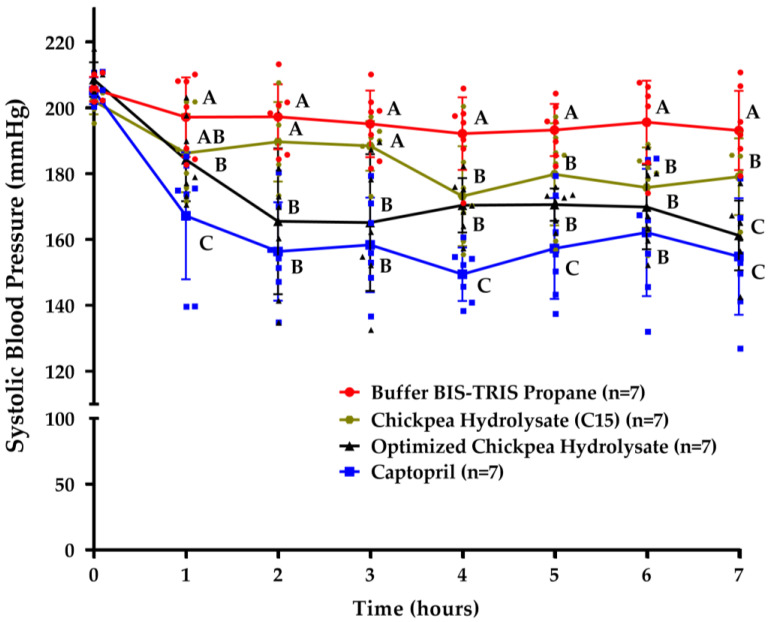
Systolic blood pressure in spontaneously hypertensive rats after supplementation with BIS-TRIS Propane buffer (20 mM, 1 mL), optimized chickpea protein hydrolysate (50 mg/kg of body weight), captopril (25 mg/kg of body weight), and chickpea protein hydrolysate (C15) (50 mg/kg of body weight). Data are presented as mean and standard deviation. Differences among SBP values across groups were determined using factorial ANOVA followed by a Benjamini, Krieger, and Yekutieli two-stage linear step-up procedure. Different letters across treatments indicate statistical differences (*p* < 0.05). Red dots (BIS-TRIS Propane buffer group), black triangles (optimized chickpea hydrolysate group), blue squares (captopril group), and green hexagons (chickpea hydrolysate C15 group) represent individual SBP values for each rat evaluated.

Figure 5 shows the SBP assessments in the supplemented SHRs (BIS-TRIS Propane buffer (negative control), OCPH, captopril (positive control), or CPH15). Compared to the negative control, the OCPH and CPH15 groups had a significantly reduced SBP at 2 h and 4 h post-supplementation, respectively (*p* < 0.05) (Figure 5). Captopril significantly reduced the SBP at all the times evaluated (*p* < 0.05). In general, the OCPH group showed statistically lower SBP values than the CPH15 group 2, 3, and 7 h post-supplementation (*p* < 0.05). Compared to the basal SBP values (time 0), the OCPH and CPH15 groups had their SBP reduced from −24.35 (11.67% (1 h)) to −47.35 mmHg (22.69% (7 h)) and from −12.41 (6.14% (2 h)) to −28.95 mmHg (14.32% (4 h)), respectively. The lowest SBP values were reached at 4 h and 7 h for the CPH15 (173.18 ± 15.19 mmHg) and OCPH (161.26 ± 10.60 mmHg) groups, respectively. The OCPH group showed SBP values similar to the captopril one at 6 h (169.92 ± 12.85 vs. 162.14 ± 19.36 mmHg, respectively) and 7 h (161.26 ± 10.60 vs. 154.86 ± 17.69, respectively) post-supplementation (*p* > 0.05).

## 4. Discussion

Some in vitro studies have shown that chickpea protein hydrolysates obtained with different enzymes can inhibit ACE-I [9,15,16]. In this context, alcalase (EC 3.4.21.62) has been used to hydrolyze proteins from different sources to obtain hydrolysates with ACE-I inhibitory potential [10,12,17]. This enzyme is a serine endopeptidase with broad hydrolysis specificity, but the uncharged amino acid residues in the P1 region are its main targets [18]. In the present study, RSA was utilized to obtain an optimized alcalase-based chickpea protein hydrolysate with the potential to inhibit ACE-I. Additionally, the hydrolysate’s hypotensive effect was evaluated in vivo. RSA allows for not only a reduction in the number of experiments needed to optimize a process but also the determination of the influence of different process variables on a given response variable [19].

The results show that hydrolysis time and temperature significantly influence the ACE-I inhibitory potential of alcalase-based chickpea protein hydrolysates. The lower the hydrolysis time, the higher the ACE-I inhibitory potential. It should be noted that the effect of hydrolysis time on ACE-I inhibition mainly depends on the enzyme used and the protein source to be hydrolyzed. For example, to produce alcalase-based amaranth or soy protein hydrolysates with the potential to inhibit ACE-I, the optimal hydrolysis time is around 6.0 h [20,21]. However, and in line with our findings, alcalase-based milk or rapeseed protein hydrolysates reduce in their ACE-I inhibitory potential as the hydrolysis time increases [22,23]. This hydrolysis time effect was previously suggested by others using a pH-stat system, and in line with our findings, they reported an optimal hydrolysis time of 30 min [14]. However, in the present study, a hydrolysis time of 5 min was enough to produce an alcalase-based chickpea protein hydrolysate with ACE-I inhibitory potential similar to the potential of the optimized hydrolysate (55.69% vs. 56.26%, respectively). Overall, the results suggest that in alcalase-based hydrolysis systems, some ACE-I inhibitory peptides released in the first 30 min can become targets of the enzyme, losing their ACE-I inhibitory capacity.

As mentioned above, one of the best alcalase-based chickpea protein hydrolysates was generated with a hydrolysis time of 5 min. Since no one has reported the production of bioactive hydrolysates with a similar hydrolysis time, SDS-PAGE was performed to determine the electrophoretic profile of such a hydrolysate, OCPH, and the chickpea protein. The electrophoretic profile of the chickpea protein isolate coincides with the different subunits of chickpea proteins [24]. Notably, a sweep of proteins was observed in the lanes loaded with the chickpea protein hydrolyzed at different times. These results confirm that alcalase hydrolyzes chickpea protein fractions and generates a peptide profile with ACE-I inhibitory potential, even at hydrolysis times as short as 5 min. This characteristic can be advantageous since it involves less time and cost to produce a hydrolysate with antihypertensive potential. Additionally, hydrolysates generated with a low degree of hydrolysis have desirable physicochemical characteristics, such as adequate gelatinization, foaming, and emulsifying capacities, among others [25]. However, dipeptides, tripeptides, and some small oligopeptides are expected to become bioavailable and to have the highest ACE-I inhibitory activity [26]. Therefore, in vivo assessments are desirable to know the antihypertensive potential of the chickpea protein hydrolysate generated after 5 min of hydrolysis with alcalase.

The hydrolysis temperature depends on the optimal working range of the enzyme used. Alcalase has the ability to hydrolyze proteins in a wide temperature range (35–75 °C) [18]. Our data indicate that hydrolysis at a temperature higher than 50 °C negatively impacts the ACE-I inhibitory potential of the hydrolysate. In fact, in the present study, the optimum hydrolysis temperature was 40 °C, although 50 °C also generated hydrolysates with more than 50% ACE-I inhibitory potential. Similarly, others reported a decrease in the ACE-I inhibitory potential of the protein hydrolysates as the temperature increased [27,28]. It should be highlighted that the temperature required to produce protein hydrolysates with the optimum ACE-I inhibitory potential depends on the protein source and enzyme utilized. For instance, hydrolysates from different food sources (e.g., flower crab meat, rapeseed, lemon seed, and amaranth) obtained at the highest temperatures evaluated showed a higher ACE-I inhibitory potential than those obtained at the lowest temperatures [20,23,29,30]. In the case of alcalase-based chickpea protein hydrolysates, in vitro studies have reported hydrolysis temperatures ≤50 °C to obtain hydrolysates with ACE-I inhibitory potential [10,14,17].

Alcalase is active at a wide range of pHs (5–11), and this property can be used to improve the ACE-I inhibitory potential of alcalase-based protein hydrolysates. Alcalase-based chickpea protein hydrolysates with the potential to inhibit up to 38% of the activity of ACE-I can be produced using conditions similar to those reported in the present study, except pH (50 °C, 30 min, enzyme/substrate 0.3 U/g, and pH 7.0) [14]. Notably, our protein hydrolysates could inhibit the activity of ACE-I by up to 56%. This increase can be attributed to the pH (11.0) of the buffer solution utilized for hydrolysis with alcalase, which could influence the pattern of the peptides released and their ACE-I inhibitory capacity.

The IC_50_ found for OCPH was 0.3583 mg/mL, which is higher than that of other chickpea protein hydrolysates reported. The IC_50_ of some protein hydrolysates with ACE-I inhibitory potential ranges from 0.28 mg/mL to 0.83 mg/mL [15,16,31,32,33,34,35], and some studies have reported antihypertensive effects in spontaneously hypertensive rats after hydrolysate supplementation [31,32,33,34]. Interestingly, hydrolysates with higher IC_50_ values than other hydrolysates from the same source can reduce blood pressure more efficiently in spontaneously hypertensive rats [32]. In general, IC_50_ is a good indicator of the potential of a protein hydrolysate to inhibit ACE-I in vitro, but in vivo assays are essential to corroborate at the physiological level its antihypertensive effect.

In the present study, OCPH and CPH15 showed similar ACE-I inhibitory activity in vitro. However, the in vivo hypotensive effect of OCPH was better than that of CPH15. Although bioavailability issues are important, these results suggest that OCPH peptides could control systolic blood pressure through mechanisms beyond ACE-I inhibition [36]. Others have suggested that antihypertensive peptides may promote vasodilation through cyclo-oxygenase and prostaglandin receptor upregulation [37]. Furthermore, it is possible that bile acid signaling exerts a hypotensive effect, but the relationship between antihypertensive peptides and bile acids remains to be explored. The first approaches to the mechanisms underlying the hypotensive effect of chickpea peptides could be based on in silico studies once the peptide sequences are elucidated [38]. Regarding in vivo approaches, evaluation of the expression of genes relevant to blood pressure control can provide insights into the mechanisms underlying the hypotensive effect of CPHs. The vasoconstrictor and vasodilator axes of the renin–angiotensin–aldosterone system are important targets for exploring hypotensive mechanisms.

SBP was efficiently reduced in the SHRs 2 and 4 h post-supplementation of OCPH and CPH15, respectively. Compared with the negative control group, the non-significant SBP reduction in the CPH15 group in the first three hours post-supplementation can be attributed to the presence of a limited number of easily absorbed ACE-I inhibitory peptides. Peptides longer than 16 amino acids can inhibit ACE-I [39], but dipeptides and tripeptides are more easily absorbed at the intestinal level than larger ones and consequently are more bioavailable [26,40]. The antihypertensive effect lasted for at least 7 h post-supplementation, which highlights the sustained hypotensive effect of the chickpea hydrolysates. This effect was up to −47.35 mmHg and −28.95 mmHg for OCPH and CPH15, respectively. Others have reported hypotensive effects that range from −9.8 mmHg to −30.45 mmHg using protein hydrolysates from different sources, different supplementation schemes, and different time points for SBP evaluations [41,42,43,44]. Notably, we recently reported that chickpea protein hydrolysates can reduce SBP in SHRs [9]. The hydrolysates were produced following sequential digestion with pepsin and pancreatin and reduced SBP by up to −61.41 mmHg [9]. To our knowledge, the present study is the first to optimize the hydrolysis conditions to produce a chickpea protein hydrolysate with ACE-I inhibitory potential and evaluate its hypotensive effect in vivo. Although OCPH was less effective in reducing SBP in the SHRs (−47.35 mmHg) than the chickpea hydrolysate obtained in a previous study (−61.41 mmHg) [9], the OCPH dose utilized in the present study (50 mg/kg) was 23-fold lower than the one used in the previous work (1200 mg/kg) [9]. In fact, the OCPH dose utilized in the present study is 0.1-0 to 47-fold lower than the doses utilized in other studies, which produced the hydrolysates from food sources other than chickpea [41,42,43,44,45]. This information suggests that both OCPH and CPH15 can efficiently reduce SBP in SHRs at convenient doses and are promising sources of ingredients for the development of functional foods or oral adjunct therapies to aid in controlling blood pressure. For instance, the human equivalent dose for OCPH is 567.67 mg for a 70 kg patient [46]. This dose is lower than that reported in clinical trials that utilize hydrolysates from sources other than chickpea [47]. Overall, our findings indicate that OCPH and CPH can promote a sustained hypotensive effect in vivo at convenient oral doses and could be incorporated into food matrices for functional food development.

## 5. Conclusions

The present study reports the hydrolysis conditions required to produce an optimized alcalase-based chickpea protein hydrolysate with ACE-I inhibitory potential. The conditions involve short hydrolysis times and 40 °C and 50 °C temperatures. The hydrolysates evaluated in vivo were OCPH and CPH15, and both efficiently reduced SBP in the SHRs for at least 7 h post-supplementation. These hydrolysates can be produced using short hydrolysis times, and their hypotensive effect can be observed at convenient oral doses. Thus, OCPH and CPH15 are promising ingredients for functional food development and potential test materials for clinical trials.

## Figures and Tables

**Table 1:** Factors and coded levels employed to optimize chickpea protein hydrolysis conditions with alcalase.

Factors	Coded Levels		
	−1	0	1
Enzyme/substrate concentration (U/g)	0.2	0.3	0.4
Time (h)	0.5	1.5	2.5
Temperature (°C)	40	50	60

**Table 2:** ACE-I inhibition of chickpea hydrolysates generated under different hydrolysis conditions with alcalase.

Conditions *	Time (h)	Enzyme/Substrate Concentration (U/g)	Temperature (°C)	ACE-I Inhibition (%) **
C2	2.5	0.2	60	39.03 ± 3.31 ^a^
C5	2.5	0.4	60	42.54 ± 4.97 ^abc^
C1	1.5	0.1583	50	44.28 ± 2.26 ^ab^
C3	1.5	0.441	50	44.47 ± 2.79 ^ab^
C4	0.5	0.2	60	44.77 ± 2.16 ^ab^
C8 (Central)	1.5	0.3	50	46.03 ± 2.0 ^b^
C7	1.5	0.3	64	46.60 ± 3.46 ^bc^
C6	2.91	0.3	50	46.87 ± 1.12 ^b^
C10	2.5	0.4	40	48.27 ± 8.50 ^abcde^
C11	2.5	0.2	40	49.06 ± 5.67 ^abcde^
C13	1.5	0.3	36	49.31 ± 3.64 ^bcde^
C9	0.5	0.4	60	49.40 ± 2.53 ^bc^
C12	0.5	0.4	40	51.31 ± 1.81 ^cd^
C14	0.5	0.2	40	54.79 ± 2.10 ^de^
C15	0.08	0.3	50	55.69 ± 3.14 ^e^
Optimized	0.5	0.2543	40	56.26 ± 0.85 ^e^

* Four replicates were considered for each condition. ** The data are presented as the mean and standard deviation. Differences across conditions were determined using Brown–Forsythe ANOVA, followed using a Benjamini, Krieger, and Yekutieli two-stage linear step-up procedure. Different letters across ACE-I inhibition values indicate statistical differences (*p* < 0.05).

## Data Availability

The original contributions presented in the study are included in the article/Supplementary Materials, further inquiries can be directed to the corresponding authors.

## Supplementary Materials

The following supporting information can be downloaded at https://www.mdpi.com/article/10.3390/foods13081216/s1. Table S1: Experimental design of alcalase hydrolysis conditions generated using response surface analysis and ACE-I inhibition of chickpea hydrolysates; Table S2: ANOVA for the effect of the processing variables on ACE-I inhibition.
