# Supplementary material for: Alcalase-Based Chickpea (Cicer arietinum L.) Protein Hydrolysates Efficiently Reduce Systolic Blood Pressure in Spontaneously Hypertensive Rats
Source: Foods. 2024 Apr 16;13(8):1216. doi: 10.3390/foods13081216 (PMC11049421; doi:10.3390/foods13081216)
Supplement: Supplementary file 1 [file foods-13-01216-s001.zip › foods-2960126-supplementary.pdf]

Supplementary Table S1. Experimental design of alcalase hydrolysis conditions generated by response surface analysis and ACE-I inhibition of chickpea hydrolysates.

Std	Run	Factor 1, A: Time (h)	Factor 2, B: Enzyme/Sustrate Concentration (U/g)*	Factor 3, C: Temperature (°C)	Response Variable ACE-I inhibition (%)
21	1	1.5	0.158393	50	44.79
31	2	1.5	0.3	50	45.11
11	3	2.5	0.2	60	41.43
8	4	2.5	0.4	40	47.65
7	5	2.5	0.4	40	48.86
20	6	2.91607	0.3	50	46.16
24	7	1.5	0.441607	50	42.25
30	8	1.5	0.3	50	44.32
18	9	0.08393	0.3	50	57.79
29	10	1.5	0.3	50	47.02
28	11	1.5	0.3	64.1607	45.34
25	12	1.5	0.3	35.8393	49.49
32	13	1.5	0.3	50	45.54
13	14	0.5	0.4	60	48.86
12	15	2.5	0.2	60	36.65
26	16	1.5	0.3	35.8393	49.14
4	17	2.5	0.2	40	44.24
33	18	1.5	0.3	50	48.28
22	19	1.5	0.158393	50	43.77
15	20	2.5	0.4	60	41.31
3	21	2.5	0.2	40	53.88
14	22	0.5	0.4	60	49.92
19	23	2.91607	0.3	50	47.34
2	24	0.5	0.2	40	53.21
9	25	0.5	0.2	60	46.52
1	26	0.5	0.2	40	56.38
6	27	0.5	0.4	40	51.76
17	28	0.08393	0.3	50	55.80
10	29	0.5	0.2	60	43.01
23	30	1.5	0.441607	50	44.29
27	31	1.5	0.3	64.1607	47.86
5	32	0.5	0.4	40	50.87
16	33	2.5	0.4	60	43.78

* A total of 10 μL of chickpea protein hydrolysate (10 mg/mL) was used for the inhibition assays.

Supplementary Table S2. ANOVA for the effect of processing variables on ACE-I inhibition.

**Source**	**Sum of Squears**	**df**	**Mean Squear**	***F*-value**	***p*-value**
Model	550.7561	8	68.8445	11.3186	< 0.0001
A- Time	210.9852	1	210.9852	34.6878	< 0.0001
B- Enzyme/Sustrate Concentration	0.9704	1	0.9704	0.1595	0.6931
C- Temperature	165.6406	1	165.6406	27.2328	< 0.0001
AB	0.6054	1	0.6054	0.0995	0.7551
AC	3.5782	1	3.5782	0.5883	0.4506
BC	38.5489	1	38.5489	6.3378	0.0189
A2	76.7910	1	76.7910	12.6251	0.0016
B2	40.8865	1	40.8865	6.7221	0.0160
Residual	145.9776	24	6.0824		
Lack of Fit	53.7229	6	8.9538	1.7470	0.1674
Pure Error	92.2547	18	5.1253		
Cor Total	696.7337	32			
